# A pilot study of a novel portable mass spectrometer for rapid, simultaneous detection of multiple anesthetic drug concentrations

**DOI:** 10.3389/fvets.2026.1776326

**Published:** 2026-02-27

**Authors:** Xiaoyu Xie, Xiaoxiao Li, Wensheng Zhang

**Affiliations:** 1 Department of Anesthesiology, West China Hospital, Sichuan University, Chengdu, Sichuan, China; 2 Laboratory of Anesthesia and Critical Care Medicine, National-Local Joint Engineering Research Centre of Translational Medicine of Anesthesiology, West China Hospital, Sichuan University, Chengdu, Sichuan, China

**Keywords:** anesthetic drugs, etomidate, lidocaine, mass spectrometer, plasma concentration, rapid analysis, rocuronium bromide

## Abstract

In the perioperative management of emergency animals, anesthetic drug–related toxicity and adverse effects pose substantial risks, and real-time quantification of anesthetic drug concentrations may provide an effective strategy for improving anesthetic safety. This study aimed to evaluate the feasibility and accuracy of a novel Cell portable mass spectrometer (MS) for the rapid detection of multiple anesthetic drug concentrations. Etomidate (ET, 1.5 mg/kg), rocuronium bromide (ROC, 5 mg/kg), and lidocaine (LID, 2 mg/kg) were administered, and plasma samples from rats were analyzed using the Cell portable MS and compared with results obtained by high-performance liquid chromatography with mass spectrometry (HPLC–MS). The Cell portable MS enabled simultaneous quantitative analysis of all three anesthetic drugs within 4.5 min and demonstrated good linearity across detection ranges. The regression equations were y = 0.01496x + 0.3136 for ET (*R*^2^ = 0.997), y = 1,356.03x + 12,785.42 for ROC (*R*^2^ = 0.991), and y = 0.01459x + 0.0067 for LID (*R*^2^ = 0.999). Pearson correlation analysis revealed strong correlations between Cell portable MS and HPLC–MS measurements for all three drugs (ET: *r* = 0.9666; ROC: *r* = 0.9858; LID: *r* = 0.9937; all *p* < 0.0001). Bland–Altman analysis showed small mean biases, with most data points falling within the 95% limits of agreement and no proportional bias observed. Overall, the Cell portable MS demonstrated good quantitative agreement with conventional HPLC–MS for rapid, simultaneous measurement of multiple anesthetic drug concentrations, supporting its potential for real-time anesthetic drug toxicity monitoring and individualized dosing, particularly in emergency settings.

## 1 Introduction

In the perioperative management of emergency animals, anesthetic drug-related toxic and adverse reactions are one of the most challenging risks (1). Emergency animals often suffer from severe trauma, underlying diseases, or unstable physiological conditions, which may substantially alter the pharmacokinetics and pharmacodynamics of anesthetic drugs (2). This heightened susceptibility increases the likelihood of dose-dependent toxic reactions, such as delayed emergence, residual neuromuscular blockade, circulatory suppression, and even local anesthetic systemic toxicity (LAST) (3). However, anesthetic drug administration is still primarily based on experience or human medicine guidelines, making it difficult to accurately reflect the individual’s actual drug exposure level in real time (4). This leads to a certain degree of “blind” anesthesia management, thereby increasing the risk of perioperative complications and mortality. Therefore, from the perspective of toxicity prevention and control, technologies that can rapidly and accurately quantify anesthetic drug concentrations in the body may become a key breakthrough in improving the safety of emergency anesthesia.

Rapid blood drug concentration monitoring offers a new approach to address the issues mentioned above (5). By providing real-time information on the actual concentration of anesthetic drugs in the body, anesthesiologists can potentially identify abnormal exposure before toxic reactions occur, allowing for timely dose adjustments or interventions to prevent dangerous consequences caused by drug accumulation, delayed metabolism, or interactions (6, 7). More importantly, this monitoring method can support individualized dosing strategies, enabling more precise control of sedation, analgesia, and muscle relaxation, which is expected to significantly reduce anesthesia risks in emergency settings (8). Therefore, developing a rapid, accurate, and bedside-applicable method for detecting anesthetic drug concentrations holds important exploratory value for the prevention of toxicity and adverse effects (9).

In recent years, portable and miniaturized mass spectrometers (MS) have emerged as a promising approach for rapid drug detection (10). These platforms integrate ambient or *in situ* ionization techniques, enabling rapid analysis of target compounds in biological samples without complex sample preparation or chromatographic separation, thereby offering distinct advantages in point-of-care testing (POCT) scenarios (11, 12). Compared with conventional liquid chromatography–mass spectrometry (LC–MS), portable MS systems offer clear benefits in terms of analysis speed, operational simplicity, and lower dependence on laboratory infrastructure, making them particularly suitable for emergency care, bedside monitoring, and resource-limited settings (13). However, despite these advantages, current portable MS technologies still face limitations related to quantitative accuracy, control of matrix effects, and the separation of complex multicomponent samples (14).

The Cell portable MS is a novel analytical platform based on in-situ ionization and ion trap MS technologies. It is compact in size and capable of rapid identification of specific compounds within approximately 1 minute, thereby supporting the possibility of real-time quantification of anesthetic drug concentrations (15). Our research group has previously demonstrated the feasibility and effectiveness of this platform for detecting a single anesthetic agent and constructing individualized pharmacokinetic–pharmacodynamic (PK–PD) models, highlighting its potential application in anesthetic toxicity monitoring (16). Given that clinical anesthesia commonly involves the combined use of multiple agents, and that metabolic and pharmacodynamic interactions among different drugs may further amplify toxicity and adverse risks (17), the objective of this study was to evaluate the feasibility and analytical performance of the Cell portable MS for rapid, simultaneous quantification of etomidate (ET), rocuronium bromide (ROC), and lidocaine (LID). We hypothesized that the Cell portable MS would provide quantitative results highly correlated and in good agreement with those obtained using conventional HPLC–MS, while offering a shorter analysis time suitable for precision anesthesia management in emergency settings (18).

## 2 Materials and methods

### 2.1 Ethics approval

This study used adult male Sprague–Dawley (SD) rats weighing 270–300 g, purchased from Dossy Technology Co., Ltd. (Chengdu, China). Animals were housed under standard laboratory conditions with controlled temperature (22 °C–25 °C) and humidity (50%–60%) under a 12 h light/dark cycle, with free access to food and water. All animal experimental procedures were conducted in accordance with the guidelines for the care and use of laboratory animals and were approved by the Animal Ethics Committee of West China Hospital, Sichuan University (approval number: 20250910005), with all efforts made to minimize animal suffering and reduce the number of animals used.

### 2.2 Animals and experimental design

Anesthesia was induced by placing the rats in an induction chamber with 5% isoflurane delivered in oxygen at a fresh gas flow of 1.0 L/min until loss of the righting reflex was achieved. After induction, anesthesia was maintained with 2% isoflurane via inhalation. Tracheal intubation was then performed, and the rats were connected to a small-animal mechanical ventilator (RWD, Shenzhen, China) for controlled ventilation. A 24-gauge intravenous catheter (Surflo Flash, Terumo, Japan) was inserted into the left femoral artery for serial blood sampling, while a separate 24-gauge catheter was placed in the tail vein for intravenous drug administration. Anesthetic doses were selected to approximate clinically relevant dosing, with ET (1.5 mg/kg), ROC (5 mg/kg), and LID (2 mg/kg) administered sequentially. Blood samples were collected at predefined time points (1, 3, 5, 10, 20, and 30 min) after drug administration for plasma concentration analysis. Given the rapid *in vivo* metabolism of ET, plasma concentration measurements for ET were performed only up to 20 min post-administration.

### 2.3 Cell portable MS detection

The Cell portable MS (C3001-sci, Suzhou Purspec Technology, Suzhou, China) is a tandem mass spectrometry–based analytical platform capable of selective precursor ion fragmentation and multistage mass spectrometric analysis of compounds, including isomers. The system consists of an ion source, ion transmission module, linear ion trap, ion detection unit, and integrated control system. The instrument measures 333 × 235 × 146 mm, weighs less than 8.5 kg, and supports a mass range of 50–1,000 m/z with switchable positive and negative ion modes. Detailed procedures for instrument calibration and quality control are provided in Supplementary Figure S1 and the Supplementary material.

MS detection was performed in positive ion mode. Quantification of ET and LID was conducted using an internal standard (IS) method. For ET, the precursor ion was m/z 245 and the fragment ion was m/z 141, with ET impurity C used as the IS (precursor ion m/z 259, fragment ion m/z 155.1). For LID, the precursor ion was m/z 235.1 and the fragment ion was m/z 86.1, with LID-d10 used as the IS (precursor ion m/z 245.2, fragment ion m/z 96.2). For ROC, the precursor ion was m/z 529.4 and the fragment ion was m/z 487.3. Although verapamil was initially evaluated as an IS, it exhibited pronounced matrix effects in the Cell MS, which could compromise quantitative accuracy; therefore, ROC was quantified using an external standard method. For analysis, A 100 μL of whole blood sample was collected without centrifugation and mixed with 400 μL of acetonitrile. For ET and LID, whole blood samples were mixed with acetonitrile containing the IS. Subsequently, 100 μL of the resulting mixture was transferred into the direct capillary spray reagent kit (Suzhou Purspec Technology, Suzhou, China). Each sample was analyzed using multiple consecutive scans in the Cell portable MS, with a total acquisition time of approximately 4.5 min per sample.

### 2.4 HPLC-MS detection

High-performance liquid chromatography with mass spectrometry (HPLC–MS, Agilent 1,260, Japan) was used as the reference method for quantitative analysis. Chromatographic separation was achieved on an Ultimate XB-C4 column using gradient elution with 0.1% formic acid aqueous solution (A) and acetonitrile (B) at a flow rate of 0.3 mL/min. Detection was performed on a triple quadrupole MS equipped with an electrospray ionization source operated in positive ion mode and multiple reaction monitoring (MRM). Plasma samples were processed by protein precipitation with acetonitrile containing the IS, followed by centrifugation and injection of the supernatant. Calibration curves covered ranges of 2–1,000 ng/mL for ET and LID and 10–10,000 ng/mL for ROC. Detailed analytical conditions are provided in the Supplementary material. Representative HPLC–MS chromatograms demonstrating adequate chromatographic separation of ET, ROC, and LID are shown in Supplementary Figure S2. Concentrations determined by HPLC–MS were used as reference values for comparison with Cell portable MS measurements.

### 2.5 Analytical methodology validation

Analytical method validation was conducted in accordance with the principles of the International Council for Harmonization (ICH) guidelines, with a focus on key performance characteristics relevant to this preliminary proof-of-concept study. Method specificity, linearity, limit of detection (LOD), and limit of quantification (LOQ) were evaluated to assess the feasibility of the Cell portable MS for rapid quantification of multiple anesthetic drugs.

### 2.6 Statistical analysis

This study was designed as a preliminary feasibility study; therefore, a small sample size (*n* = 3 rats) was used. Statistical analysis was performed using GraphPad Prism software (Version 10.4, Boston, USA). Data were presented descriptively, with individual values shown for each animal. The standard curves were fitted by linear regression models. For ET and LID, the theoretical concentrations were used as the independent variable (x), and the IS–corrected peak area ratios were used as the dependent variable (y). For ROC, the theoretical concentration was used as the independent variable (x), and the peak intensity was used as the dependent variable (y). Considering the greater variability in ROC responses at low concentration levels, a weighted least-squares regression with a 1/x weighting factor was applied to improve fitting stability, whereas ordinary least-squares linear regression was used for ET and LID. The goodness of fit was evaluated using the coefficient of determination (*R*^2^), with *R*^2^ ≥ 0.99 indicating good linearity.

Pearson correlation analysis was performed to assess the linear relationship between anesthetic drug concentrations measured by the Cell portable MS and HPLC–MS, with the correlation coefficient (*r*) and its statistical significance were calculated. An *r* value > 0.95 was considered to indicate an extremely strong correlation, reflecting high consistency in measurement trends between the two methods (19).

Agreement between the two platforms was further evaluated using Bland–Altman analysis. The percentage difference (%Difference) between measurements obtained by the Cell portable MS and HPLC–MS was plotted on the *y*-axis against the mean on the *x*-axis, and the 95% limits of agreement (LoA) were calculated. Agreement was considered acceptable when most data points fell within the 95% LoA and no proportional bias was observed across the concentration range (20).

## 3 Results

### 3.1 Specificity

To evaluate the analytical specificity of the Cell portable MS for ET, ROC, and LID, MS analyses were performed using blank and standard blood samples, and the corresponding full-scan mass spectra are shown in Supplementary material. For ET (Supplementary Figure S3), no interfering signals were observed at m/z 228.0 in blank samples, whereas a distinct ET peak was detected at the same m/z in standard samples. Similarly, no background interference was detected at the characteristic ions of ROC (m/z 487.2; Supplementary Figure S4) or LID (m/z 85.9; Supplementary Figure S5) in blank samples, while clear and well-resolved signals were observed in standard samples. These results demonstrated that the Cell portable MS enabled reliable identification of ET, ROC, and LID based on their characteristic ions and exhibited good analytical specificity in complex blood matrices.

### 3.2 Linear range

**Figure 1:**
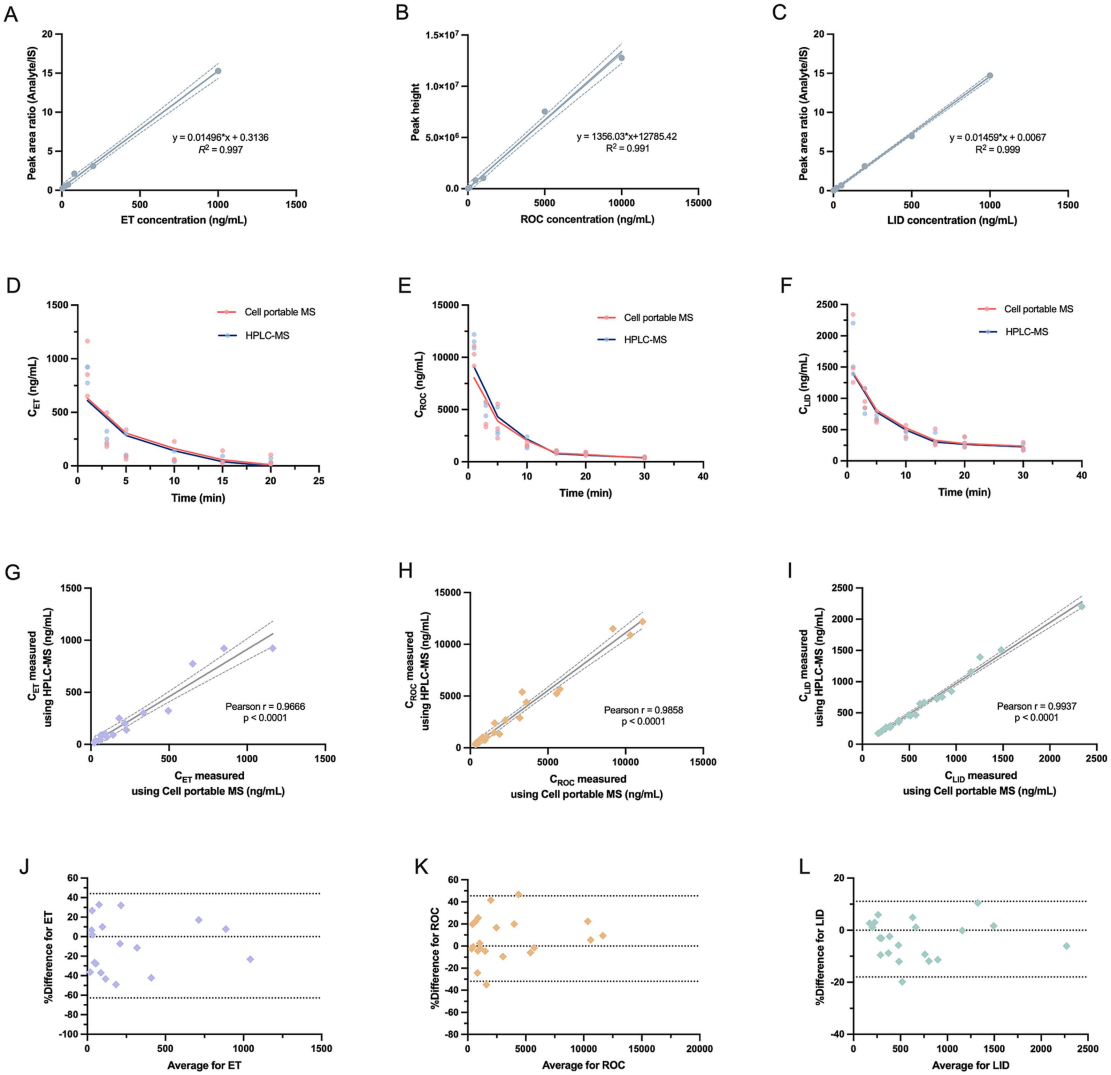
Comparison of multiple anesthetic drug concentrations (ET, ROC, and LID) measured by the Cell portable MS and HPLC–MS. **(A–C)** Linear range of ET, ROC, and LID measured by the Cell portable MS. Each data point represents a standard sample at a defined concentration level. **(D–F)** Concentration-time profiles measured by the Cell portable MS and HPLC–MS. Individual data points represent plasma concentrations from each animal, and solid lines show smoothed curves to illustrate overall concentration–time trends. **(G–I)** Correlation analysis for plasma concentrations of ET, ROC, and LID measured by the Cell portable MS and HPLC–MS. Each data point represents an individual plasma sample collected from each rat at predefined time points. **(J–L)** Bland–Altman agreement plots of the three drugs. Data points represent plasma concentrations obtained from each rat at predefined time points. ET, etomidate; ROC, rocuronium bromide; LID, lidocaine.

Standard curves were established using a series of standard samples across graded concentration levels. For ET (Figure 1A), calibration was performed over concentrations of 2, 4, 8, 20, 40, 80, 200, and 1,000 ng/mL, yielding a regression equation of y = 0.01496x + 0.3136 with an *R*^2^ of 0.997. For ROC (Figure 1B), weighted linear regression (1/x) was applied across concentrations of 10, 20, 50, 100, 500, 1,000, 5,000, and 10,000 ng/mL, resulting in the equation y = 1,356.03x + 12,785.42 with an *R*^2^ value of 0.991. For LID (Figure 1C), calibration over concentrations of 2, 4, 10, 20, 50, 200, 500, and 1,000 ng/mL produced a regression equation of y = 0.01459x + 0.0067 with an *R*^2^ of 0.999. Detailed regression parameters, including the standard deviation of the slope and intercept and the residual standard deviation, are provided in the Supplementary Table S1. Overall, all three anesthetic drugs demonstrated good linearity within their respective tested concentration ranges.

### 3.3 LOD and LOQ

The LOD and LOQ for the three anesthetic drugs were determined using a signal-to-noise (S/N) approach. For ET, the LOD was 49.35 ng/mL at an S/N ratio of 3, and the LOQ was 213.39 ng/mL at an S/N ratio of 10. Similarly, the LOD and LOQ for ROC were 9.63 ng/mL and 54.10 ng/mL, respectively, while those for LID were 1.36 ng/mL and 5.63 ng/mL. Detailed calculation procedures are provided in the Supplementary material.

### 3.4 Consistency comparison

Figures 1D–F show the concentration–time curves for the three drugs measured by Cell portable MS and HPLC–MS. The peak concentrations and overall concentration–time trends of ET, ROC, and LID were consistent between the two platforms, in accordance with their expected *in vivo* pharmacokinetic behavior. Further Pearson correlation analysis (Figures 1G–I) demonstrated strong correlations in the measured concentrations of all three anesthetic drugs between the two analytical platforms, with correlation coefficients close to 1.0 (ET: *r* = 0.9666; ROC: *r* = 0.9858; LID: *r* = 0.9937) and all *p* values < 0.0001. These results indicated a high degree of consistency in the measurement trends of drug concentration changes between the two instruments.

Bland–Altman analysis was then conducted to evaluate the agreement between the two platforms (Figures 1J–L). The mean biases for ET, ROC, and LID were −9.38%, 6.77%, and −3.44%, respectively, indicating relatively small overall bias. For all three drugs, most points fell within the 95% LoA, and no proportional bias was observed across the tested concentration ranges. These results indicated that the Cell portable MS exhibited good quantitative agreement in the measurement of plasma concentrations of ET, ROC, and LID, with results showing good comparability to those obtained using conventional HPLC–MS.

## 4 Discussion

Rapid measurement of anesthetic drug plasma concentrations represents an important strategy for preventing drug-related toxicity. In this study, ET, ROC, and LID were selected as representative anesthetic agents to evaluate the application potential of a novel Cell portable MS for the simultaneous monitoring of multiple anesthetic drugs. The results demonstrate that this system enables quantitative analysis of these three drugs within minutes and effectively reflects overall trends in drug concentrations, providing a technical basis for dynamic assessment of *in vivo* anesthetic drug exposure.

Anesthetic management often requires the combined use of multiple agents, including sedatives, neuromuscular blocking agents, and analgesics, to achieve adequate anesthetic depth, muscle relaxation, and analgesia. However, the administration of multiple drugs also significantly increases the risk of drug-related toxicity. ET is a commonly used intravenous sedative; however, excessive or repeated administration may lead to adrenal suppression (21). ROC may result in residual neuromuscular blockade or respiratory depression due to its substantial interindividual variability (22, 23). LID can induce central nervous system and cardiac toxicity particularly at high doses or under impaired metabolism conditions (24). These adverse effects are more likely to be amplified in emergency, trauma, or physiologically unstable patients, thereby increasing the risk of drug-related toxicity. Therefore, monitoring approaches capable of rapid, simultaneous detection of multiple anesthetic drugs and timely assessment of dynamic drug exposure are crucial for perioperative management, particularly in emergency anesthesia settings (25).

The agreement analysis showed that the mean biases for all three drugs were relatively small, and most points fell within the 95% LoA, with no evident proportional bias observed. These findings suggested that the Cell portable MS exhibited stable quantitative performance across different concentration ranges. It is noteworthy that the width of the LoA varied among the three drugs, which may be attributable to differences in their physicochemical properties, matrix effects, and ionization efficiencies (26, 27). Given that this study was designed as a small-sample, proof-of-concept investigation, with the primary objective of evaluating the feasibility of the Cell portable MS platform for rapid multi-drug detection rather than defining strict clinically acceptable error thresholds, the observed differences remain within a reasonable and interpretable range. In addition, as part of the methodological evaluation, preliminary assessments of precision and accuracy were performed and are presented in the Supplementary material, indicating that the Cell portable MS exhibits acceptable reproducibility and quantitative accuracy, supporting its potential for further translational development.

Beyond analytical performance, the Cell portable MS platform also demonstrates advantages from the perspective of Green Analytical Chemistry (GAC) (28). Its compact and lightweight design eliminates the need for high-power vacuum systems and complex chromatographic separation modules, thereby reducing energy demands. In addition, the ability to perform rapid, multi-drug analysis significantly shortens analysis time, which may help lower overall energy consumption throughout the analytical process. By requiring minimal plasma volumes and avoiding complex pretreatment, the Cell portable MS reduces solvent and sample consumption and has the potential to decrease laboratory chemical waste over long-term application.

This study has several limitations. For ROC, external calibration was used because the tested IS exhibited pronounced matrix effects on the Cell portable MS platform. Although good linearity and agreement with HPLC–MS were achieved, more systematic evaluation of ion suppression and matrix effects would further enhance quantitative robustness. In addition, this study assessed simultaneous quantification of only three commonly used anesthetic drugs, whereas clinical anesthesia often involves multiple agents; therefore, future work should extend this approach to a broader range of anesthetics. Finally, as a pilot study with a limited number of animals, the present findings primarily demonstrate feasibility and relative analytical performance and warrant validation in larger-scale studies.

## 5 Conclusion

In summary, the Cell portable MS enables rapid measurement of anesthetic drug plasma concentrations, offering a practical approach for toxicity prevention and individualized dosing. By providing real-time concentration information, this platform has the potential to support more precise anesthetic management and reduce the risk of drug-related adverse effects. With further methodological optimization and validation in larger samples, the Cell portable MS may be extended to other anesthetics and perioperative drugs, contributing to improved anesthesia safety and rational drug use in emergency and resource-limited settings.

## Data Availability

The original contributions presented in the study are included in the article/Supplementary material, further inquiries can be directed to the corresponding author.

## References

[1] Brodbelt D. Perioperative mortality in small animal anaesthesia. Vet J. (2009) 182:152–61. doi: 10.1016/j.tvjl.2008.06.011, 18658000

[2] Aguilera R Sinclair M Valverde A Bateman S Hanna B. Dose and cardiopulmonary effects of propofol alone or with midazolam for induction of anesthesia in critically ill dogs. Vet Anaesth Analg. (2020) 47:472–80. doi: 10.1016/j.vaa.2020.03.006, 32402602

[3] Sasaki K Rabozzi R Kasai S Ikeda K Ishikawa T. Fentanyl-induced muscle rigidity in a dog during weaning from mechanical ventilation after emergency abdominal surgery: a case report. Vet Med Sci. (2023) 9:37–42. doi: 10.1002/vms3.1001, 36409227 PMC9857132

[4] Pinho RH Nasr-Esfahani M Pang DSJ. Medication errors in veterinary anesthesia: a literature review. Vet Anaesth Analg. (2024) 51:203–26. doi: 10.1016/j.vaa.2024.01.003, 38570267

[5] Krishnakumar A Mishra RK Kadian S Zareei A Rivera UH Rahimi R. Printed graphene-based electrochemical sensor with integrated paper microfluidics for rapid lidocaine detection in blood. Anal Chim Acta. (2022) 1229:340332. doi: 10.1016/j.aca.2022.340332, 36156230

[6] Riff C Le Caloch A Dupouey J Allanioux L Leone M Blin O . Local anesthetic plasma concentrations as a valuable tool to confirm the diagnosis of local anesthetic systemic toxicity? A report of 10 years of experience. Pharmaceutics. (2022) 14:35456542. doi: 10.3390/pharmaceutics14040708, 35456542 PMC9025106

[7] Fang Z Zhang H Guo J Guo J. Overview of therapeutic drug monitoring and clinical practice. Talanta. (2024) 266:124996. doi: 10.1016/j.talanta.2023.124996, 37562225

[8] Coetzee E Absalom AR. Pharmacokinetic and pharmacodynamic changes in the older adults: impact on Anesthetics. Clin Geriatr Med. (2025) 41:19–35. doi: 10.1016/j.cger.2024.03.004, 39551539

[9] Kuo FH Tudor BH Gray GM Ahumada LM Rehman MA Watkins SC. Precision Anesthesia in 2050. Anesth Analg. (2024) 138:326–36. doi: 10.1213/ane.0000000000006688, 38215711

[10] Liu X Wang B Luo H Zou J Yang BC Hu B. Portable miniature mass spectrometry for enhanced on-site detection of analytes in complex samples by integrating solid-phase microextraction and Nano-electrospray ionization. Anal Chem. (2024) 96:17471–5. doi: 10.1021/acs.analchem.4c04224, 39415685

[11] Zhang Q Zhu X Li J Zhang Y Wang C Ma Q. Nanomaterial-enhanced ambient ionization for miniature mass spectrometry: toward high-performance on-site detection. Mikrochim Acta. (2025) 192:392. doi: 10.1007/s00604-025-07238-2, 40455291

[12] Zhou D Wu J Wang Q Liu Y Wang S Zhang W . Miniature mass spectrometry for point-of-care testing the GPIIb/IIIa inhibitor tirofiban during perioperative period of percutaneous coronary intervention. ACS Omega. (2024) 9:50326–33. doi: 10.1021/acsomega.4c06581, 39741813 PMC11683618

[13] Smith BL Hankinson T Maher S. Portable instrumentation for ambient ionization and miniature mass spectrometers. Annu Rev Anal Chem (Palo Alto, Calif). (2024) 17:69–102. doi: 10.1146/annurev-anchem-061522-040824, 38640067

[14] Ma X Ouyang Z. Ambient ionization and miniature mass spectrometry system for chemical and biological analysis. Trends Anal Chem. (2016) 85:10–9. doi: 10.1016/j.trac.2016.04.009, 28042191 PMC5193165

[15] Zhang S Li L Xie Y Fan L Wang Y Wang N . Rapid identification and on-site analysis by miniature mass spectrometry of chemical markers for fragrant rosewood authentication. J Pharm Biomed Anal. (2025) 252:116490. doi: 10.1016/j.jpba.2024.116490, 39393212

[16] Li X Chang P Liu X Gong D Zhang W. Rapid quantification and PK-PD modeling of rocuronium bromide in beagles using portable mass spectrometer. Front Vet Sci. (2025) 12:1543086. doi: 10.3389/fvets.2025.1543086, 40104546 PMC11914140

[17] Brown EN Pavone KJ Naranjo M. Multimodal general Anesthesia: theory and practice. Anesth Analg. (2018) 127:1246–58. doi: 10.1213/ane.0000000000003668, 30252709 PMC6203428

[18] Fei Q Zhang Y Liu C Zheng J Fu Q. Artificial intelligence in anesthesia and perioperative medicine. Anesthesiol Perioper Sci. (2025) 3:24. doi: 10.1007/s44254-025-00107-4

[19] Schober P Boer C Schwarte LA. Correlation coefficients: appropriate use and interpretation. Anesth Analg. (2018) 126:1763–8. doi: 10.1213/ane.0000000000002864 29481436

[20] Giavarina D. Understanding bland altman analysis. Biochem Med Zagreb. (2015) 25:141–51. doi: 10.11613/bm.2015.015, 26110027 PMC4470095

[21] Zhang H Wu A Nan X Yang L Zhang D Zhang Z . The application and pharmaceutical development of etomidate: challenges and strategies. Mol Pharm. (2024) 21:5989–6006. doi: 10.1021/acs.molpharmaceut.4c00325, 39495089

[22] Naguib M Brull SJ Kopman AF Hunter JM Fülesdi B Arkes HR . Consensus statement on perioperative use of neuromuscular monitoring. Anesth Analg. (2018) 127:71–80. doi: 10.1213/ane.0000000000002670, 29200077

[23] Pedersen K Kruhøffer LL Lykkesfeldt J Kousholt BS. Comparison of the neuromuscular effects of two infusion rates of rocuronium in anesthetized pigs. Acta Vet Scand. (2022) 64:38. doi: 10.1186/s13028-022-00658-7, 36522634 PMC9753331

[24] Foo I Macfarlane AJR Srivastava D Bhaskar A Barker H Knaggs R . The use of intravenous lidocaine for postoperative pain and recovery: international consensus statement on efficacy and safety. Anaesthesia. (2021) 76:238–50. doi: 10.1111/anae.15270, 33141959

[25] Arroyo-Currás N Ortega G Copp DA Ploense KL Plaxco ZA Kippin TE . High-precision control of plasma drug levels using feedback-controlled dosing. ACS Pharmacol Transl Sci. (2018) 1:110–8. doi: 10.1021/acsptsci.8b00033, 32219207 PMC7088981

[26] Keller BO Sui J Young AB Whittal RM. Interferences and contaminants encountered in modern mass spectrometry. Anal Chim Acta. (2008) 627:71–81. doi: 10.1016/j.aca.2008.04.043, 18790129

[27] Wang J Pursell ME DeVor A Awoyemi O Valentine SJ Li P. Portable mass spectrometry system: instrumentation, applications, and path to 'omics analysis. Proteomics. (2022) 22:e2200112. doi: 10.1002/pmic.202200112, 36349734 PMC10278091

[28] Tobiszewski M Mechlińska A Namieśnik J. Green analytical chemistry--theory and practice. Chem Soc Rev. (2010) 39:2869–78. doi: 10.1039/b926439f, 20502819

