# Supplementary material for: A pilot study of a novel portable mass spectrometer for rapid, simultaneous detection of multiple anesthetic drug concentrations
Source: Front Vet Sci. 2026 Feb 27;13:1776326. doi: 10.3389/fvets.2026.1776326 (PMC12982080; doi:10.3389/fvets.2026.1776326)
Supplement: Supplementary file 1 [file Supplementary_file_1.docx]

Supplementary Material

# Calibration and Quality Control of the Cell Portable MS

With respect to calibration and quality control of the Cell portable MS, several measures were implemented in this study. As shown in **Supplementary Figure S1**, prior to each day’s analysis, the instrument was routinely calibrated using a positive-ion mode calibration kit to ensure accurate mass axis alignment, and corresponding calibration and performance reports were generated to support analytical stability and reliability. In addition, the instrument undergoes regular performance maintenance and status evaluation approximately every six months to maintain optimal operating conditions.


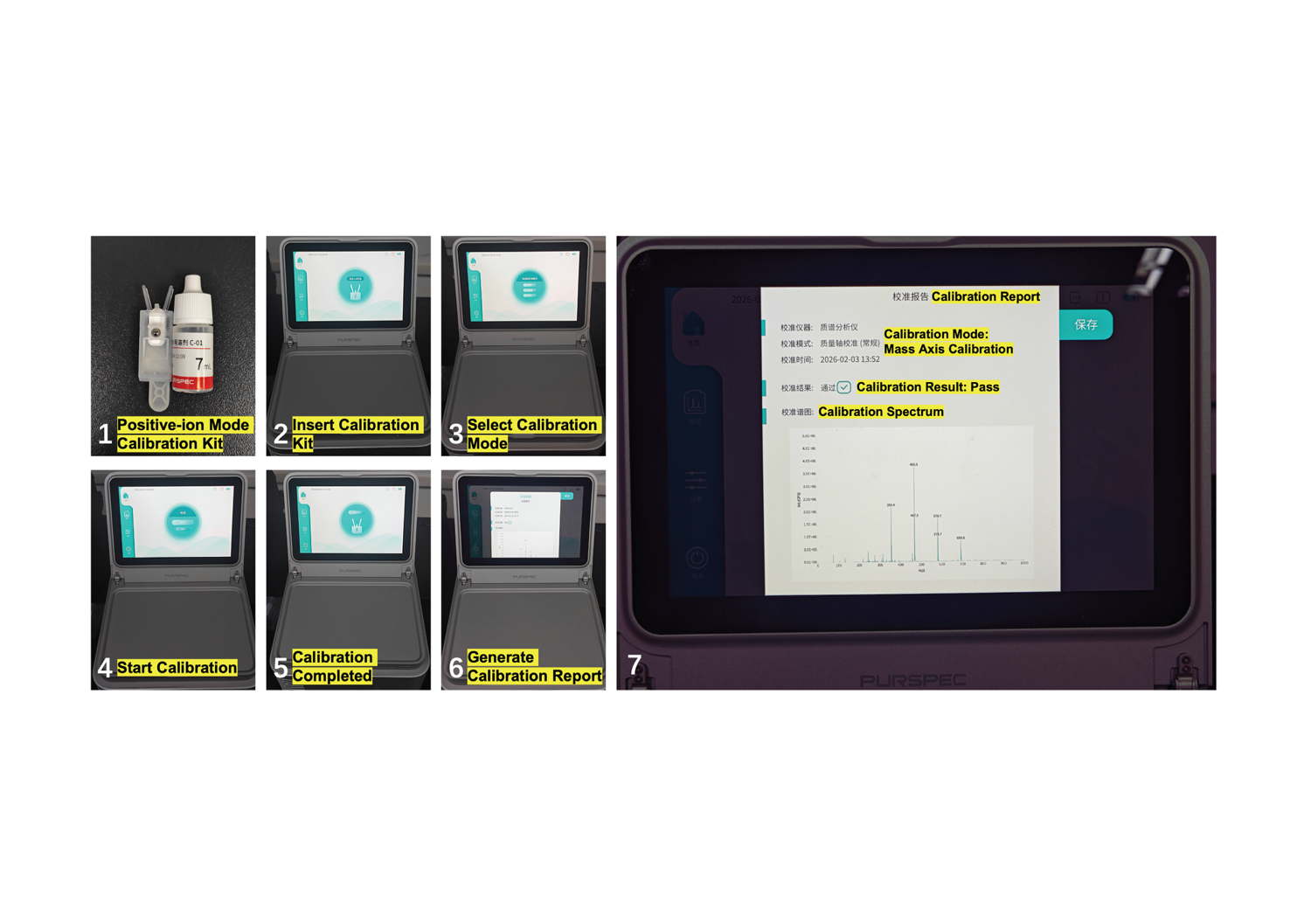


**Supplementary Figure S1** Quality calibration procedure of the Cell portable MS. The procedure includes insertion of the positive-ion mode calibration kit, selection of the calibration mode, initiation and completion of calibration, generation of the calibration report, and evaluation of calibration results. The calibration spectrum and a “pass” result indicate acceptable mass accuracy and instrument performance prior to analytical measurements.

# HPLC-MS Detection for Etomidate (ET), Rocuronium Bromide (ROC), and Lidocaine (LID)

## Instruments and Reagents

The analytical system consisted of an Agilent 1260 high-performance liquid chromatography system coupled to an Agilent G6460 triple quadrupole mass spectrometer operated with the Agilent MassHunter workstation (Agilent Technologies). A refrigerated high-speed centrifuge (Allegra 64R, Beckman Coulter, USA), an analytical balance with 0.01 mg readability (Sartorius ME215s), and a vortex mixer (IKA MS3 basic) were used for sample preparation. Acetonitrile (HPLC grade; Noersh), formic acid (HPLC grade; Aladdin), and ultrapure water (Watsons) were used throughout the study.

## Chromatographic Conditions

Chromatographic separation was performed on an Ultimate XB-C4 column (3.0 mm × 100 mm i.d., 3 μm, 300 Å; Welch Materials). The mobile phase consisted of solvent A (0.1% formic acid aqueous solution) and solvent B (acetonitrile). The flow rate was maintained at 0.3 mL/min. The gradient program was as follows: 25% B held for 2 min; increased linearly from 25% to 60% B over 2–4 min; increased to 90% B at 4.1 min. The total run time was 7 min, followed by a 2.5-min post-run equilibration. The column temperature was maintained at 30°C, and the injection volume was 1 μL.

## Mass Spectrometric Conditions

Mass spectrometric detection was performed using an electrospray ionization (ESI) source operated in positive ion mode with multiple reaction monitoring (MRM). The drying gas temperature was set at 350°C with a gas flow rate of 5 L/min. The nebulizer pressure was 45 psi. The sheath gas temperature was 350°C with a flow rate of 11 L/min. The capillary voltage was set at 3,500 V.

The MRM transitions and parameters were as follows:

- Rocuronium bromide: m/z 529.4 → 487.3; fragmentor voltage 134 V; collision energy 32 V
- Verapamil (internal standard for rocuronium): m/z 455.3 → 165.2; fragmentor voltage 150 V; collision energy 28 V
- Etomidate: m/z 245 → 141; fragmentor voltage 80 V; collision energy 4 V
- ET impurity C (internal standard for etomidate): m/z 259 → 155.1; fragmentor voltage 85 V; collision energy 0 V
- Lidocaine: m/z 235.1 → 86.1; fragmentor voltage 115 V; collision energy 16 V
- Lidocaine-d10 (internal standard for lidocaine): m/z 245.2 → 96.2; fragmentor voltage 115 V; collision energy 16 V

## Sample Preparation and Analysis

Plasma samples (50 μL) were mixed with 150 μL of internal standard solution (acetonitrile containing 20 ng/mL verapamil, 10 ng/mL lidocaine-d10, and 25 ng/mL ET impurity C), vortex-mixed, and centrifuged at 20,000 rpm for 10 min at 4°C. The supernatant was transferred into autosampler vials and analyzed by HPLC–MS. The linear concentration ranges were 2–1,000 ng/mL for etomidate and lidocaine, and 10–10,000 ng/mL for rocuronium bromide.

# Representative HPLC–MS Chromatograms of ET, ROC, and LID

#
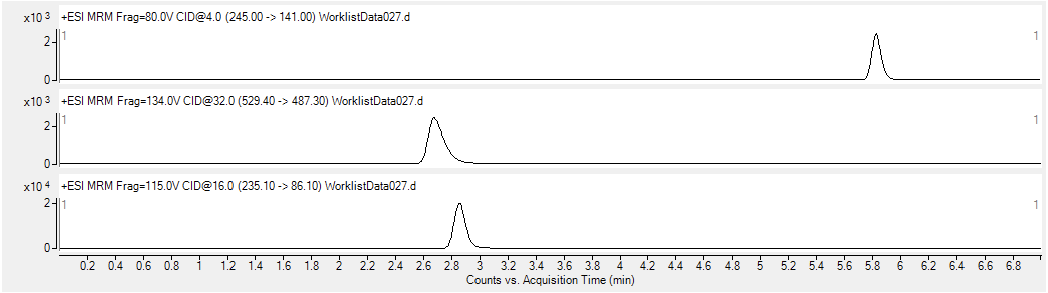


**Supplementary Figure S2** Representative HPLC–MS chromatograms of ET, ROC, and LID in rat plasma. Chromatograms demonstrated adequate chromatographic separation of ET, ROC, and LID under the established HPLC–MS conditions, with no evident interference from endogenous plasma components.

# Full-scan Mass Spectra of ET, ROC, and LID Measured by the Cell Portable MS


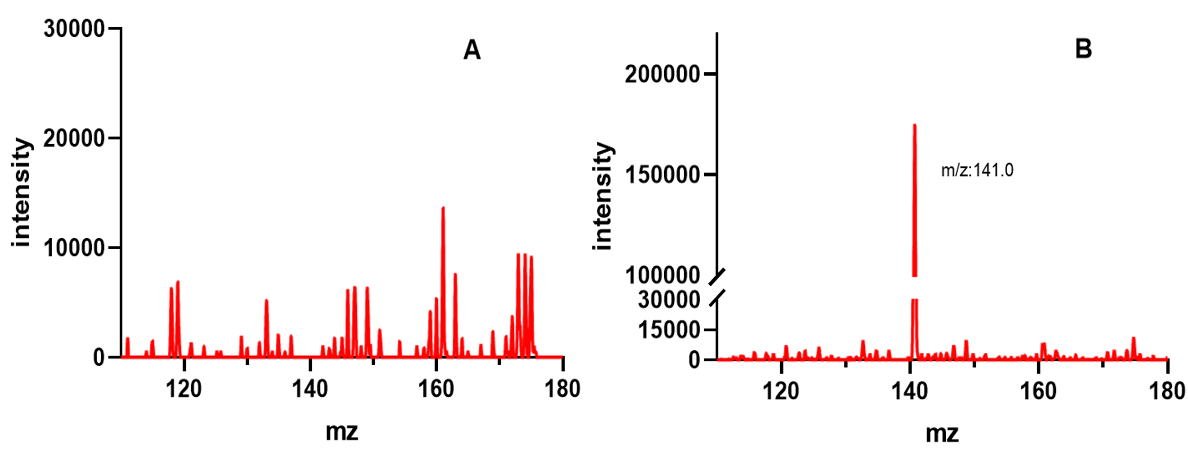


**Supplementary Figure S3** Full-scan mass spectra of ET measured by Cell portable MS. **(A)** Full-scan mass spectrum of blank rat plasma; **(B)** full-scan mass spectrum of rat plasma spiked with ET, showing a clear characteristic signal at m/z 141.0.


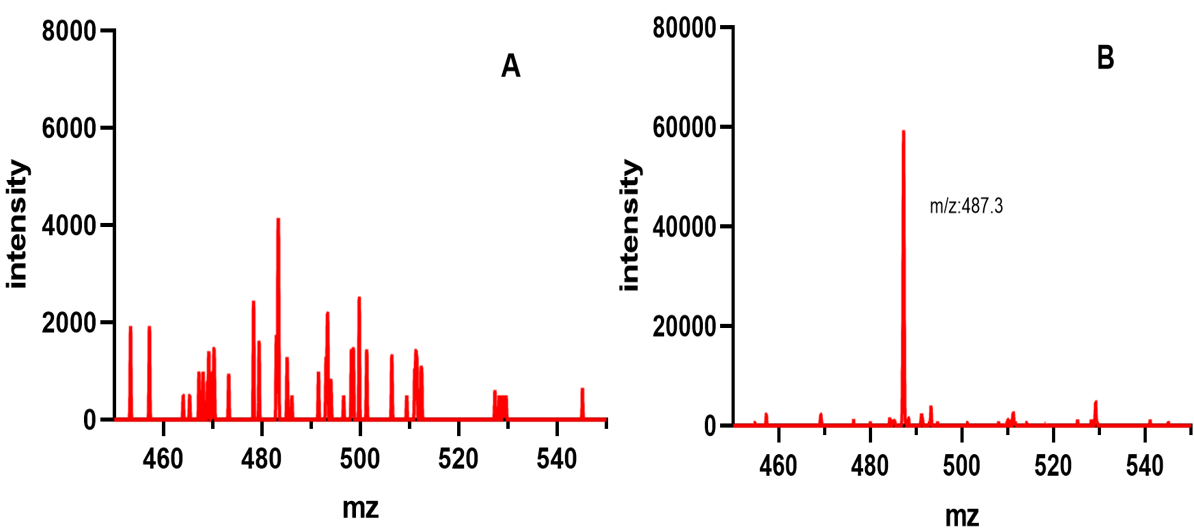


**Supplementary Figure S4** Full-scan mass spectra demonstrating the analytical specificity of the Cell portable MS for ROC. **(A)** Full-scan mass spectrum of blank rat plasma; **(B)** full-scan mass spectrum of rat plasma spiked with ROC, with a distinct characteristic ion observed at m/z 487.3.


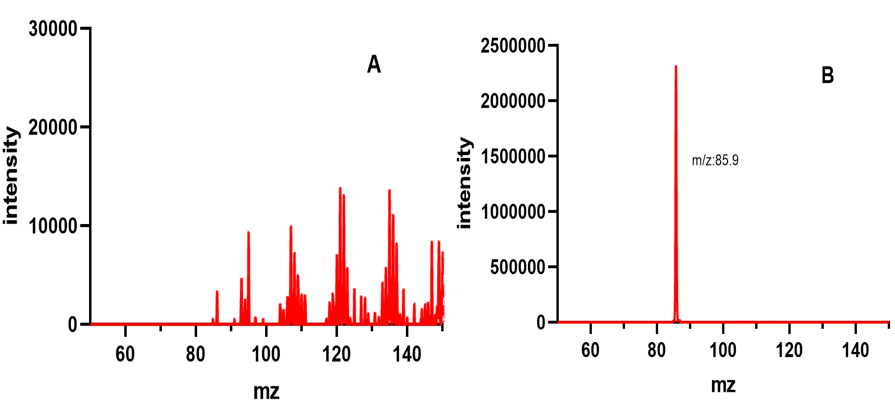


**Supplementary Figure S5** Full-scan mass spectra of LID measured by Cell portable MS. **(A)** Full-scan mass spectrum of blank rat plasma; **(B)** full-scan mass spectrum of rat plasma spiked with LID, showing a specific signal at m/z 85.9.

# Regression Parameters of Standard Curves Measured by the Cell Portable MS

**Supplementary Table S1** Regression parameters of standard curves for ET, ROC, and LID measured by the Cell portable MS.

| Analyte | Slope SD | Intercept SD | Residual SD |
| --- | --- | --- | --- |
| ET | 0.000404 | 0.169 | 0.349 |
| ROC | 57.59 | 17,371.93 | 7,359 |
| LID | 0.000178 | 0.072 | 0.168 |

ET, etomidate; ROC, rocuronium bromide; LID, lidocaine; SD, standard deviation.

# Calculation of LOD and LOQ

The limits of detection (LOD) and quantification (LOQ) were determined based on signal-to-noise ratios of 3 and 10, respectively, using regression equations derived from the standard curves.

For ET, the mean background noise measured at m/z 141.0 in blank rat plasma was 5,796, and the mean signal intensity of the internal standard (ET impurity C) was 16,531. Based on the regression equation y = 0.01496x + 0.3136, the LOD and LOQ were determined to be 49.35 ng/mL and 213.39 ng/mL, respectively.

For ROC, the mean background noise at m/z 487.3 in blank plasma was 8,614. Using the weighted calibration equation (1/x), y = 1,356.03x + 12,785.42, the LOD and LOQ were 9.63 ng/mL and 54.10 ng/mL, respectively.

For LID, the mean background noise at m/z 85.9 was 18,994, and the mean signal intensity of the internal standard LID-d10 was 2,136,759. According to the regression equation y = 0.01459x + 0.0067, the LOD and LOQ were calculated as 1.36 ng/mL and 5.63 ng/mL, respectively.

# Precision and Accuracy

Precision and accuracy were assessed using quality control samples prepared at two concentration levels (low and high): ET at 40 and 200 ng/mL, ROC at 500 and 5,000 ng/mL, and LID at 20 and 500 ng/mL. At each concentration level, three replicate samples were analyzed under identical conditions. Precision was expressed as the relative standard deviation (RSD, %), reflecting inter-assay variability, while accuracy was evaluated based on recovery rates. For ET, RSD ranged from 10.60% to 19.49%, with accuracy between 87.93% and 112.89%. For ROC, RSD values ranged from 18.75% to 19.73%, and accuracy ranged from 86.60% to 116.51%. For LID, RSD ranged from 1.14% to 12.84%, with accuracy between 93.62% and 105.24%. Overall, these results demonstrated acceptable precision and accuracy for all three analytes within the tested concentration ranges, supporting the feasibility of the Cell portable MS for rapid quantitative analysis.
